# Improving Annatto Residue Bioconversion for *Pleurotus ostreatus* var. Florida Cultivation via Supplementation Strategies

**DOI:** 10.3390/microorganisms14071405

**Published:** 2026-06-25

**Authors:** Milton Mineo Hirai, Lucas da Silva Alves, Wagner Gonçalves Vieira Junior, Marcos Antônio Da Silva Freitas, Pedro Afonso Gomes Teixeira, Adriano Taffarel Camargo De Paula, Isabela Vitória De Paula Moretti, Diego Cunha Zied

**Affiliations:** 1 Departamento de Produção Vegetal, Faculdade de Ciências Agrárias e Tecnológicas, Universidade Estadual Paulista (UNESP), Dracena 17900-000, Brazil; mineo.hirai@unesp.br (M.M.H.); marcos.freitas@unesp.br (M.A.D.S.F.); isabela.p.moretti@unesp.br (I.V.D.P.M.); 2 Programa de Pós-Graduação em Microbiologia Agropecuária, Faculdade de Ciências Agrárias e Veterinárias, Universidade Estadual Paulista (UNESP), Jaboticabal 14.884-900, Brazil; silva.alves@unesp.br (L.d.S.A.); afonso.gomes@unesp.br (P.A.G.T.); adriano.taffarel@unesp.br (A.T.C.D.P.); 3 Pós-Graduação em Sociedade, Tecnologia e Meio Ambiente, Universidade Evangélica de Goiás, Anápolis 75083-515, Brazil; vieira.jr@unesp.br; 4 Sedmo—Solos, Ecologia e Dinâmica da Matéria Orgânica, Centro Universitário Evangélico de Goianésia, Goianésia 76380-000, Brazil

**Keywords:** bioeconomy, biological efficiency, lignocellulosic biomass, mushroom, substrate formulation

## Abstract

The valorization of agro-industrial residues is essential for advancing circular bioeconomy systems. This study integrated the natural colorant and edible mushroom industries by evaluating annatto (*Bixa orellana*) residues as substrates for *Pleurotus ostreatus* cultivation. Two experiments were conducted, testing field and industrial residues at three incorporation levels (32.5%, 42.5%, and 52.5%, *w*/*w* on a dry weight basis) combined with different supplementation strategies (corn bran, wheat bran, and their mixture) in a completely randomized design. Field residues showed greater yield and biological efficiency, while industrial residues exhibited higher variability. Total yield reached 38.92%, while the lowest value was 24.28%, representing an increase of up to 65% depending on residue origin and supplementation strategy. Biological efficiency exceeded 140% under optimal conditions, with gains above 70% compared to the lowest-performing treatments. Field residues also promoted a higher number of bunches and greater average bunch mass. Overall, substrate origin, supplementation, and residue proportion were decisive for fungal performance, demonstrating that annatto residues are promising low-cost substrates for scalable mushroom production within circular economy systems.

## 1. Introduction

Global mushroom production has expanded rapidly over recent decades, increasing more than 30-fold since the late 20th century [1] and reaching over 40 million tons annually, with projections exceeding 50 million tons in the coming years [2].

Among cultivated species, *Pleurotus* spp. has emerged as one of the most important groups, accounting for approximately 20% of global production and showing continuous market growth driven by its adaptability, low production cost, and ability to utilize lignocellulosic residues [2,3]. In this context, *Pleurotus ostreatus* var. Florida, commonly known as the white oyster mushroom, belongs to the group of white-rot fungi and is characterized by an efficient enzymatic system capable of degrading lignin, cellulose, and hemicellulose through the production of oxidative and hydrolytic enzymes. This metabolic versatility enables the conversion of complex agro-industrial residues into edible biomass with high nutritional value [4].

The use of diverse lignocellulosic residues is recognized as an effective strategy to enhance productivity and agronomic performance in *Pleurotus* cultivation. Oyster mushrooms can colonize a wide range of agro-industrial residues, which serve as sources of carbon, nitrogen, and essential minerals [5]. While cellulose and hemicellulose serve as major carbohydrate sources for fungal metabolism, lignin acts as a structural barrier that limits biomass accessibility. White-rot fungi such as *Pleurotus* spp. can degrade lignin and efficiently utilize structural carbohydrates, enhancing substrate bioconversion efficiency [6].

The use of lignocellulosic residues in different formulations promotes structural modifications in substrate components during *Pleurotus* cultivation, enhancing the degradation of cellulose and hemicellulose and improving overall substrate utilization efficiency [7]. In addition to increasing yield, the incorporation of agro-industrial residues can also influence the nutritional and sensory quality of the mushrooms, as variations in substrate composition affect the availability of nutrients and bioactive compounds during growth [8]. A wide range of agro-industrial and lignocellulosic residues, including materials derived from agricultural, agroforestry, industrial, and urban systems, have already been reported as suitable substrates for *Pleurotus* cultivation [7,8,9,10].

Despite the wide range of agro-industrial residues already explored for *Pleurotus* cultivation, several region-specific biomasses remain underutilized. Among them, annatto (*Bixa orellana* L.) has gained increasing economic importance due to its use as a natural colorant in the food, cosmetic, pharmaceutical, and textile industries [11]. The extraction of bixin, the main pigment, generates substantial amounts of residual biomass both in the field and during industrial processing [12], which are often underutilized or lack well-defined management strategies. Considering the scale of annatto production, these residues represent an important biomass resource with potential for valorization through fungal cultivation systems.

These residues present a relevant lignocellulosic composition, as annatto seeds contain significant proportions of cellulose, hemicellulose, lignin, carbohydrates, and proteins, in addition to residual bioactive compounds [12,13]. In particular, the reported cellulose content in annatto seeds (up to 40–45%) highlights their potential as a carbon-rich substrate component for mushroom production [12]. At the same time, the presence of lignin-rich fractions makes this biomass especially relevant for white-rot fungi such as *Pleurotus ostreatus* var. Florida, which can transform recalcitrant lignocellulosic materials into edible biomass. Furthermore, the growing demand for natural pigments has expanded annatto production, particularly in South America, where cultivation is largely associated with smallholder farming systems and plays an important socioeconomic role [14,15].

In this context, the valorization of annatto residues through mushroom cultivation represents a promising strategy not only for sustainable waste management but also for generating additional income for growers and strengthening food production systems under resource-limited conditions. However, the application of annatto residues as a substrate component for *Pleurotus* cultivation remains largely unexplored, highlighting a clear gap in the development of integrated and sustainable production systems.

Therefore, this study evaluates annatto (*B. orellana*) residues from field and industrial origins as substrate components for *P. ostreatus* var. Florida cultivation, assessing different residue incorporation levels and supplementation strategies to enhance bioconversion and agronomic performance.

## 2. Materials and Methods

### 2.1. Site of Experiment

The study was conducted at the Mushroom Research Center (CECOG), at the School of Agricultural and Technological Sciences (FCAT) at São Paulo State University (UNESP), Dracena campus, Brazil (21°28′57″ S, 51°33′58″ W; altitude 400 m). The experiments were carried out in controlled growth chambers, featuring a fully optimized environment with precise monitoring of O_2_, CO_2_, humidity, and luminosity for mushroom production.

### 2.2. Annatto Residue Origin and Processing

The study consisted of two independent experiments conducted under the same cultivation conditions, differing only in the origin of annatto residues (field or industrial). For each residue origin, a 3 × 3 factorial arrangement was established, combining three annatto residue proportions (32.5%, 42.5%, and 52.5%) with three supplementation strategies (corn bran, wheat bran, and corn bran + wheat bran).

The annatto waste materials used in this study were obtained from local producers through a regional company (Urucum Brasil Ltda., Monte Castelo, Brazil). The annatto residues field and industrial-derived represent distinct structural and compositional profiles. Field-derived residues were obtained directly from post-harvest conditions. Due to the semi-mechanized harvesting system commonly used for annatto, a substantial amount of crop residue remains in the field after harvest. These materials, composed mainly of husks, fragmented plant tissues, and residual seeds, were collected and used for substrate preparation in the experimental treatments [16].

**Figure 1:**
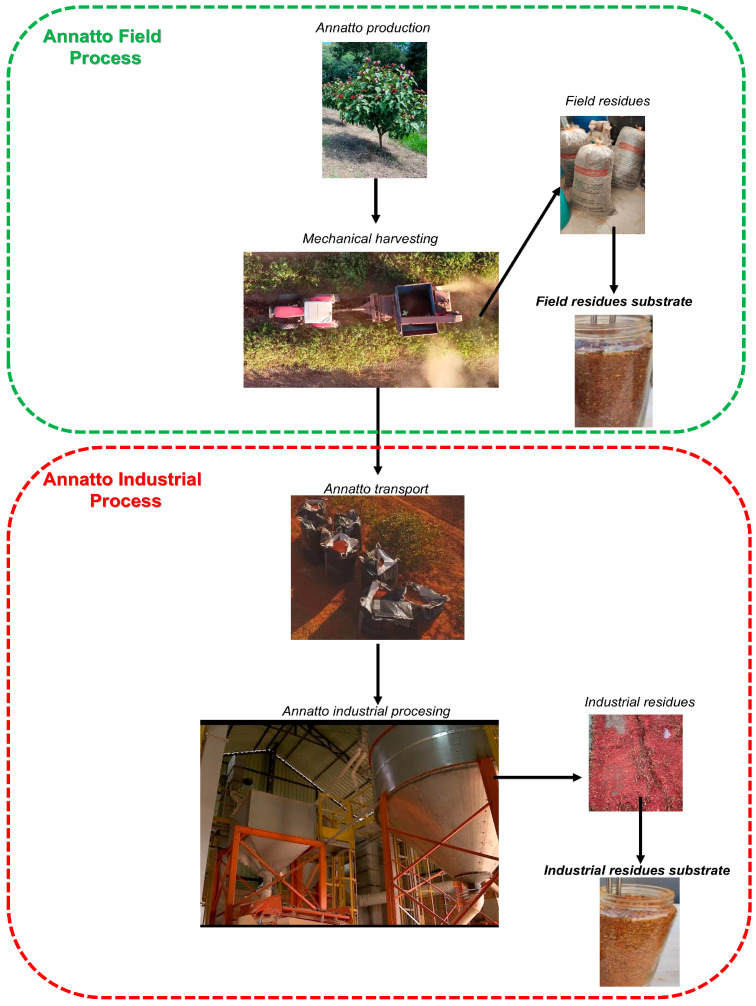
Schematic representation of field and industrial residue production during annatto processing.

In contrast, industrial residues originated from the final processing stage prior to commercialization and consisted predominantly of material retained during sieving. This fraction contained a higher proportion of annatto seeds and exhibited a more intense reddish coloration, attributable to the presence of natural pigments concentrated in the seed coat [16,17]. Figure 1 illustrates the origin of the annatto residues evaluated in this study.

### 2.3. Substrate Production and Experimental Design

After the collection of annatto residues (field and industrial), the materials were ground, sieved (5/16 mesh), and homogenized. The substrates were then formulated according to each treatment by combining annatto residues with sugarcane bagasse, wheat bran, corn bran, and calcium carbonate (CaCO_3_). Subsequently, the substrates were characterized in terms of C/N ratio, moisture content, pH, and electrical conductivity, as described in Table 1.

Substrates based on annatto residues from field and industrial sources were evaluated at three incorporation levels (32.5%, 42.5%, and 52.5%, *w*/*w* on a dry weight basis) to test a gradient of residue replacement while maintaining suitable physical conditions for substrate colonization. Corn bran, wheat bran, and their combination were used as supplementation strategies because they differ in nutrient composition and may differentially improve the nutritional balance of the substrate.

After substrate mixing and homogenization, moisture adjustment was performed according to the methodology proposed by Albertia et al. [4]. The treatments were then identified and packed according to their respective formulations into high-density polypropylene bags containing 800 g of wet substrate. The bags were pressed, sealed, and sterilized in an autoclave at 121 °C for 4 h, as described by Zied et al. [18].

The experimental design followed a completely randomized design (CRD), with each treatment consisting of 9 replicates. A total of 81 experimental units were established for field residues and 81 for industrial residues, resulting in an overall sample size of *n* = 162.

### 2.4. Spawn Production and Inoculation

The inoculum used in this study was produced according to the methodology described by Zied et al. [18], including grain preparation, pH correction with calcium carbonate (CaCO_3_), sterilization, and inoculation from a mother spawn. The strain used was *P. ostreatus* var. Florida POS 23/01 (white oyster mushroom), obtained from the culture collection of the Mushroom Research Center (CECOG), maintained in the culture collection, and first reactivated on Petri dishes containing a composite potato dextrose agar (PDA) medium. After inoculation, the Petri dishes were incubated in a BOD chamber at 25 °C for 12 days to allow full mycelial colonization. Subsequently, sorghum grains were inoculated with mycelial plugs obtained from these plates to produce the mother spawn.

The sorghum grains were prepared by boiling in water at a ratio of 1 kg of grains to 2 L of water for approximately 40 min. After cooking, the grains were drained, supplemented with 2% (*w*/*w*) calcium carbonate, packed into polypropylene bags, and sterilized by autoclaving at 121 °C for 2 h. The grains were then inoculated with the mother spawn at a rate of 2% (*w*/*w*) under aseptic conditions (16 g of spawn per bag).

### 2.5. Mushroom Production and Harvesting

After cooling and inoculation of the sterilized substrate, the inoculated bags were sealed and incubated at 26 ± 2 °C and 60% relative humidity for 20 days to allow complete mycelial colonization (spawn run). After full colonization, the plastic bags were opened by cutting the upper surface and transferred to fruiting conditions. Fruiting was induced at 22 ± 2 °C and 85 ± 5% relative humidity, following Albertia et al. [4].

Harvesting was performed twice daily over a total production period of 42 days, which was divided into three flushes.

### 2.6. Variables Analyzed

The agronomic and bioconversion performance of *P. ostreatus* var. Florida was evaluated based on quantitative variables measured throughout the cultivation cycle [19].

Harvesting was conducted over three production flushes, and all variables were recorded separately for each flush and cumulatively. The evaluated parameters included total fresh mass (g), convertibly as the yield of mushrooms harvested per experimental unit (bag); mushroom weight (g), calculated as the ratio between total fresh mass and the number of individual mushrooms; bunches mass (g), obtained by dividing total fresh mass by the number of bunches; number of mushrooms; and number of bunches per bag (Table S1).

From these primary variables, yield (%) and biological efficiency (BE, %) were calculated. The yield was determined as

(1) $$yield\;(\%)=\frac{fresh\;mushroom\;mass\;(g)}{fresh\;substrate\;mass\;(g)}\times 100$$

while the BE was calculated as

(2) $$BE\;(\%)=\frac{fresh\;mushroom\;mass\;(g)}{dry\;substrate\;mass\;(g)}\times 100$$

These indices allowed the assessment of substrate conversion efficiency and yield performance across treatments, considering both individual flush dynamics and total production.

### 2.7. Statistical Analysis

The study consisted of two independent experiments conducted under the same environmental and cultivation conditions, differing only in the origin of annatto residues (field and industrial). For each residue origin, the data were analyzed using a completely randomized design (CRD) in a 3 × 3 factorial arrangement, considering supplementation strategy (corn bran, wheat bran, and corn bran + wheat bran) and annatto residue proportion (32.5%, 42.5%, and 52.5%) as fixed factors.

Analysis of variance (ANOVA) was performed separately for each residue origin to evaluate the effects of treatments and their interactions (Table S2). When significant differences were detected (*p* ≤ 0.05), means were compared using Tukey’s honestly significant difference (HSD) test at a 5% probability level. All statistical analyses were performed using Sisvar software version 5.8 Build 92 (DES/UFLA, Lavras, Brazil).

## 3. Results

### 3.1. Yield and Biological Efficiency

During cultivation, three experimental units were discarded due to contamination, corresponding to an overall contamination rate of 1.85%. Table 2 presents the yield of the first, second, and third flushes, as well as the cumulative total yield obtained throughout the experimental period. Yield was significantly influenced by the interaction between residue type, proportion, and supplementation strategy in various stages.

We observed that annatto field residues exhibited lower variation in total yield across proportions. Specifically, total yield values of 35.03, 34.23, and 33.11% were observed at substitution levels of 32.5, 42.5, and 52.5%, respectively. In contrast, it was found that annatto industrial residues showed greater variability, with total yields of 34.12, 32.02, and 27.83% at the same substitution levels. Furthermore, it was observed that industrial residues presented a reduced productivity in the first flush.

For annatto field residues, we observed that the condition combining 32.5% residue with corn bran and wheat bran supplementation resulted in significantly superior performance. This treatment demonstrated the highest first flush yield among all treatments (25.31%) while maintaining stable second and third flushes, which resulted in the highest total yield observed in the study (38.92%).

As a more stable condition, it was found that the use of 42.5% annatto field residues supplemented with wheat bran resulted in the second-highest total yield (35.20%), which may be associated with improved performance in the second flush and reduced variation between the first and third flushes. At the highest proportion (52.5%), it was observed that a general decline in performance occurred, with only limited gains in total yield compared to other treatments.

For annatto industrial residues, we observed that the use of 32.5 or 42.5% annatto industrial residues supplementation with corn bran and wheat bran demonstrated statistically superior performance. These treatments showed the highest first flush yield among all treatments (23.33 and 22.38%, respectively), which contributed to the highest total yield (38.20 and 38.02%, respectively).

Supplementation with either corn bran or wheat bran alone under annatto industrial residue conditions resulted in lower average productivity values (29.05% and 29.12%, respectively). This outcome was associated with significantly reduced productivity during the first flush, with decreases of approximately 50% for corn bran and 22% for wheat bran compared with the combined supplementation of both brans.

It was also observed that increasing the residue proportion to 52.5% under combined supplementation (corn + wheat bran) resulted in a marked reduction in total yield (31.97%), which was mainly associated with the sharp decline observed in the first flush (11.53%).

Biological efficiency (BE) values are presented in Table 3. For annatto field residues, we observed that the 42.5% proportion resulted in the highest mean BE (166.24%), compared to 136.24% and 108.93% at 32.5% and 52.5%, respectively. Among supplementation strategies, it was found that wheat bran resulted in the highest mean BE (165.51%). Notably, the highest BE value observed for field residues was obtained under wheat bran supplementation at a 42.5% residue proportion (244.91%), which was significantly higher than all other treatments.

Although the 52.5% proportion showed the lowest overall BE (108.93%), we observed that supplementation with corn bran resulted in a higher BE (125.13%) compared to wheat bran (113.19%) and combined supplementation (88.48%) at the same proportion.

For annatto industrial residues, the 52.5% proportion resulted in the lowest BE (85.63%), indicating a negative effect of excessive residue addition. In contrast, the 42.5% and 32.5% proportions showed significantly higher BE values (103.20% and 120.70%, respectively), with significant differences also observed between these lower proportions. The highest BE values observed under industrial residue conditions were obtained with corn bran + wheat bran supplementation at 32.5 and 42.5% annatto industrial residue (showing BE of 141.51 and 146.26%), which was significantly higher than the values observed for 52.5% annatto industrial residue (89.95%).

### 3.2. Mean Mass, Number of Mushrooms and Bunches

Mean mushroom mass is presented in Table 4. We observed that, for annatto field residues, a higher average mushroom mass was obtained (1.46 g), with clear superiority under corn bran supplementation across all evaluated proportions. Specifically, corn bran resulted in values of 1.51, 1.52, and 1.37 g at 32.5%, 42.5%, and 52.5% residue proportions, respectively, consistently outperforming the other supplementation strategies.

For annatto industrial residues, we observed a trend of increasing mean mushroom mass with increasing residue proportion. The substitution levels of 32.5%, 42.5%, and 52.5% resulted in mean values of 1.23, 1.16, and 1.33 g, respectively. This effect was particularly evident under wheat bran supplementation, where we observed an increase from 1.11 g at 42.5% to 1.42 g at 52.5%, representing an increase of approximately 0.31 g per mushroom.

The number of mushrooms is presented in Table 5. For annatto field residues, we observed that wheat bran and the combined supplementation (corn bran + wheat bran) resulted in the highest number of mushrooms, with mean values of 238.35 and 241.26, respectively, whereas corn bran alone showed a lower number of mushrooms (186.24). It was verified that increasing the proportion of annatto residue tended to increase the number of mushrooms, reaching the highest mean at 52.5% (237.08).

For annatto industrial residues, we observed a different response pattern. It was demonstrated that increasing residue proportion resulted in a reduction in the number of mushrooms, with values decreasing from 214.88 at 32.5% to 188.69 at 52.5%. Among supplementation strategies, corn bran maintained the highest stability and resulted in the highest number of mushrooms at 52.5% (243.77), whereas wheat bran and the combined supplementation showed significant reductions, particularly at higher residue levels (170.22 and 147.50, respectively).

Bunches mean mass is presented in Table 6. For annatto field residues, we observed that corn bran supplementation resulted in the highest bunch mass, with a mean value of 15.23 g, significantly outperforming wheat bran (10.17 g) and the combined supplementation (12.23 g). It was verified that, at the intermediate residue proportion (42.5%), the highest bunch mass was achieved (13.59 g), mainly driven by corn bran (16.98 g).

For annatto industrial residues, we observed a different interaction pattern. It was demonstrated that the effect of supplementation depended strongly on residue proportion. Corn bran showed stable performance across all levels, maintaining bunch mass between 9.33 and 10.29 g. In contrast, wheat bran exhibited a significant reduction with increasing residue proportion, decreasing from 11.06 g at 32.5% to 7.94 g at 52.5%, indicating a negative interaction between higher industrial residue content and this supplementation. On the other hand, the combined supplementation (corn bran + wheat bran) demonstrated a compensatory effect, with a marked increase at 52.5% (10.30 g), surpassing both individual supplements at this level.

The number of bunches is presented in Table 7. For annatto field residues, we observed that wheat bran supplementation resulted in the highest number of bunches, with a mean value of 29.50, significantly outperforming corn bran (18.88) and the combined supplementation (24.69). It was verified that the highest bunch production occurred at 52.5% residue proportion (25.96), mainly driven by wheat bran, which reached 33.12 bunches.

The interaction between supplementation and residue proportion was clearly demonstrated. While wheat bran showed a positive response with increasing residue proportion, the combined supplementation (corn bran + wheat bran) exhibited a peak at 32.5% (29.12), followed by a reduction at higher proportions.

For annatto industrial residues, we observed a more stable pattern across treatments. It was demonstrated that the number of bunches showed only a slight decrease with increasing residue proportion, from 27.88 at 32.5% to 26.53 at 52.5%. Among supplementation strategies, wheat bran resulted in the highest cluster number at 42.5% (33.37), whereas corn bran showed the highest value at 52.5% (30.33). The combined supplementation remained relatively stable across all proportions, indicating a buffering effect against variations in residue composition.

## 4. Discussion

This study proposes the integration of two agro-industries—natural colorants and edible mushrooms—within a bioconversion of waste in food. Annatto (*B. orellana*) stands out as one of the most important natural colorants in the food industry due to its high technological compatibility and low toxicity [13]. However, its harvest process generates substantial amounts of lignocellulosic residues, which are rich in carbon and nutrients but remain largely underutilized [17].

The literature suggests that these by-products could be transformed into sustainable packaging materials and biodegradable films [11]; however, such approaches would significantly increase processing costs and shift the production chain from an agricultural to a more industrial model, which may not be feasible for small-scale growers to implement at the farm level.

The performance of *Pleurotus* spp. on lignocellulosic substrates is strongly influenced by substrate composition and structure. White-rot fungi such as *P. ostreatus* produce a complex enzymatic arsenal, including laccases, manganese peroxidases, versatile peroxidases, cellulases, and hemicellulases, whose abundance and activity vary according to substrate composition [6]. Previous studies have demonstrated that differences in cellulose, hemicellulose, lignin content, and substrate physical structure can alter enzyme expression patterns, substrate utilization efficiency, and ultimately mushroom productivity [20,21].

Therefore, variations in the chemical and structural characteristics of annatto residues may have influenced the degradation dynamics of the substrate and the availability of nutrients required for fungal growth and fruiting. The substrate formulations (Table 1) exhibited C/N ratios of 21.8–36.7 and pH values of 5.63–8.35, both within the ranges considered favorable for *Pleurotus ostreatus* cultivation (C/N: 20–40; pH: 5.0–9.0) [22,23]. Moisture content ranged from 62 to 85%, with most treatments close to the recommended interval of 60–75% reported for oyster mushroom production [24].

Differences in residue origin may also influence mycelial development through changes in nutrient accessibility and substrate degradation dynamics. The initial colonization phase depends on the ability of the mycelium to establish itself within the substrate and mobilize carbon sources [25]. We hypothesize that field residues may preserve a more balanced lignocellulosic matrix, with greater structural integrity and a more gradual release of nutrients, favoring fungal colonization and substrate utilization [22]. These characteristics may have contributed to the differences observed in yield, biological efficiency, and mushroom production between field and industrial residues. However, the specific physicochemical and biological factors responsible for these responses were not directly evaluated in the present study and should be investigated in future research.

In this context, the proportion of annatto residue acted as a key factor modulating substrate performance according to the supplementation strategy. Overall, field residues maintained relatively stable total yield across the different substitution levels, whereas industrial residues showed greater variability and a reduction in yield as the proportion of annatto residue increased (Table 3). This pattern may be largely explained by the responses observed during the first flush (Table 2), where a pronounced interaction between residue proportion and supplementation strategy was detected, influencing the subsequent productive performance of *Pleurotus* cultivation, as the first flush commonly accounts for a substantial proportion of total mushroom production [26,27].

At the treatment level for annatto field residues, the strong contribution of the first flush to total productivity suggests that early substrate utilization played a major role in determining cultivation performance. The superior yield observed for the combination of corn bran and wheat bran at 32.5% annatto residue was largely associated with higher first flush production (Table 2), whereas wheat bran supplementation at 42.5% resulted in a more balanced production pattern across flushes. This treatment also achieved the highest biological efficiency (244.91%) (Table 3), reinforcing the importance of wheat bran supplementation for optimizing substrate utilization and mushroom production. Wheat bran has been widely reported as an effective supplement for *Pleurotus cultivation* due to its favorable nutritional composition, particularly its contribution of nitrogen and readily available nutrients [26,28].

The industrial residues were more dependent on the supplementation strategy, and increasing annatto residue incorporation generally reduced both yield and biological efficiency (Table 2 and Table 3). The combination of corn bran and wheat bran resulted in the highest total yield (35.92%), supported by high first flush production and a consistent second flush. A similar trend was observed for biological efficiency. The superior performance of this supplementation strategy may be related to the greater nutritional diversity provided by the combination of different bran and/or materials, which could favor fungal development on agro-industrial residues [26,29]. However, increasing annatto residue incorporation to 52.5% significantly reduced total yield (27.83%) and biological efficiency (126.08%), suggesting that excessive incorporation may compromise substrate utilization efficiency, highlighting the importance of balancing residue valorization with productive performance.

From an applied perspective, these findings have important implications for the design of circular bioeconomy systems. The ability to efficiently utilize both field and industrial annatto residues depends on understanding their functional differences and adjusting substrate formulations accordingly. While field residues can be incorporated without major reductions in productivity, industrial residues require more careful optimization of the supplementation strategy and inclusion level.

Furthermore, the effects of the supplementation strategy on biomass allocation were modulated by residue origin and proportion. For annatto field residues, increasing the substitution level tended to enhance the number of mushrooms (Table 5) and the bunches (Table 7), particularly under wheat bran supplementation, indicating that higher residue proportions provided a good structural and nutritional environment for primordia formation [30,31]. However, this increase in fruiting body number was accompanied by a reduction in individual mass, reinforcing the existence of a resource allocation trade-off. In contrast, corn bran maintained higher mushroom and bunch mass across all proportions (Table 4 and Table 6), suggesting a more efficient conversion of available carbon into biomass expansion, regardless of residue level.

For annatto industrial residues, a distinct response pattern was observed. Increasing residue proportion led to a reduction in the number of mushrooms (Table 5), particularly under wheat bran and combined supplementation, indicating that higher levels of industrial residue may limit primordia initiation. Under these conditions, corn bran supplementation showed greater stability, maintaining both mushroom number and mass even at 52.5% [32]. Overall, higher proportions of industrial residue were associated with reduced numbers and mean mass of mushrooms, highlighting the importance of optimizing substrate formulation.

Therefore, annatto residue recycling should not be viewed solely as a waste management strategy, as previously proposed by other authors [11,14], but rather as a biologically relevant factor that directly influences fungal metabolism and bioconversion. In this context, substrate origin emerges as a critical variable in the design of supplementation strategies for *Pleurotus* cultivation, particularly in systems aimed at integrating agro-industrial by-products within circular economy frameworks. By demonstrating how residue origin and formulation strategies modulate fungal performance, these findings contribute to the development of more efficient, predictable, and scalable cultivation systems.

Moreover, this approach is aligned with current global regulatory trends that emphasize waste valorization, resource efficiency, and the replacement of synthetic inputs with natural and renewable alternatives, e.g., outlined in the EU Waste Framework Directive [33]. Thus, the strategic use of annatto residues represents not only a technical advancement but also a pathway toward compliance with sustainability-driven policies, reinforcing their role as functional inputs in next-generation circular bioeconomy systems.

## 5. Conclusions

Annatto residues can be efficiently utilized as substrates for *Pleurotus ostreatus* var. Florida cultivation; however, their performance is strongly dependent on residue origin and supplementation strategy. Field residues provided optimal performance, particularly with wheat bran or combined supplementation. In contrast, industrial residues showed higher variability, with better performance observed at lower residue incorporation levels under the combined corn bran and wheat bran supplementation strategy, indicating the need for more precise formulation. However, both residue types would benefit from further investigation regarding enzymatic activity, substrate degradation dynamics, and nutritional characterization to better explain the observed differences between different substrates.

## Figures and Tables

**Table 1:** Substrate formulations (%) and physicochemical composition of substrates prepared with annatto residues from field and industrial origins under different supplementation strategies.

Origin of Annatto Residues	Treatments	Substrate Formulation (*w*/*w* on a Dry Weight Basis)					Physicochemical Composition ^1^					
		Annatto Residues	Sugarcane Bagasse	Wheat Bran	Corn Bran	CaCO_3_	C (%)	N (%)	C/N Ratio	pH	E.C. (µS cm^−1^)	Moisture (%)
Field	1	32.5	32.5	-	33.0	2	49.7	1.74	28.5	6.35	1.59	74
	2	42.5	22.5	-	33.0	2	48.2	1.96	24.6	6.96	1.81	85
	3	52.5	12.5	-	33.0	2	48.9	1.98	24.7	5.79	2.70	71
	4	32.5	32.5	33.0	-	2	50.4	1.37	36.7	6.84	0.82	76
	5	42.5	22.5	33.0	-	2	48.3	1.56	30.9	6.86	0.96	75
	6	52.5	12.5	33.0	-	2	48.7	1.72	28.3	8.35	1.21	73
	7	32.5	32.5	16.5	16.5	2	47.1	1.47	32.0	6.54	1.47	73
	8	42.5	22.5	16.5	16.5	2	40.5	1.86	21.8	6.89	1.65	63
	9	52.5	12.5	16.5	16.5	2	49.9	1.39	36.0	5.63	2.32	69
Industrial	10	32.5	32.5	-	33.0	2	49.6	1.92	25.9	6.78	2.43	67
	11	42.5	22.5	-	33.0	2	49.5	2.13	23.2	6.45	2.74	73
	12	52.5	12.5	-	33.0	2	49.5	2.09	23.7	6.18	3.56	73
	13	32.5	32.5	33.0	-	2	50.3	1.59	31.7	6.92	1.31	62
	14	42.5	22.5	33.0	-	2	50.1	1.43	35.1	6.24	1.66	62
	15	52.5	12.5	33.0	-	2	50.5	1.77	28.5	6.60	2.25	73
	16	32.5	32.5	16.5	16.5	2	49.7	1.79	27.9	7.03	1.82	74
	17	42.5	22.5	16.5	16.5	2	50.5	1.68	30.1	7.11	1.67	66
	18	52.5	12.5	16.5	16.5	2	50.0	1.93	25.9	6.52	2.19	70

^1^ Moisture (before substrate autoclaving) was determined by oven drying at 105 °C until constant weight. pH and electrical conductivity (E.C.) were measured in aqueous extracts (1:10, *w*/*v*). Total carbon (C) and nitrogen (N) were determined by elemental analysis, and the C/N ratio was calculated from these values.

**Table 2:** Yield (%) of *P. ostreatus* var. Florida cultivated on annatto residues under different supplementation strategies and residue proportions for both field and industrial origins.

**Annatto field residues**																			
**1st flush**					**2nd flush**					**3rd flush**					**Total yield**				
**Supplementation**	% Annatto residue *			**Mean**	**Supplementation**	**% Annatto residue**			**Mean**	**Supplementation**	**% Annatto residue**			**Mean**	**Supplementation**	**% Annatto residue**			**Mean**
	**32.5**	**42.5**	**52.5**			**32.5**	**42.5**	**52.5**			**32.5**	**42.5**	**52.5**			**32.5**	**42.5**	**52.5**	
Corn bran	20.11 B	19.73	21.50	20.45	Corn bran	6.32 B	6.33 B	7.76	6.80 B	Corn bran	4.53	4.70	4.52	4.58 B	Corn bran	30.97 B	31.97	33.78	32.24
Wheat bran	19.56 B	20.61	18.28	19.48	Wheat bran	9.95 A	10.32 A	9.17	9.81 A	Wheat bran	5.68	5.72	5.38	5.59 A	Wheat bran	35.20 AB	36.73	32.82	34.91
Corn bran + wheat bran	25.31 Aa	20.20 b	21.50 ab	22.27	Corn bran + wheat bran	8.18 AB	8.48 AB	7.40	8.02 AB	Corn bran + wheat bran	5.42	5.31	4.03	4.91 AB	Corn bran + wheat bran	38.92 A	34.00	32.73	35.22
Mean	21.73	20.18	20.36		Mean	8.09	8.38	8.11		Mean	5.19	5.24	4.65		Mean	35.03	34.23	33.11	
C.V.	19.32				C.V.	27.21				C.V.	29.16				C.V.	20.89			
**Annatto industrial residues**																			
**1st flush**					**2nd flush**					**3rd flush**					**Total Yield**				
**Supplementation**	**% Annatto residue**			**Mean**	**Supplementation**	**% Annatto residue**			**Mean**	**Supplementation**	**% Annatto residue**			**Mean**	**Supplementation**	**% Annatto residue**			**Mean**
	**32.5**	**42.5**	**52.5**			**32.5**	**42.5**	**52.5**			**32.5**	**42.5**	**52.5**			**32.5**	**42.5**	**52.5**	
Corn bran	12.56 B	8.70 C	8.40	9.98 C	Corn bran	12.56	15.37 A	14.12 A	13.99 A	Corn bran	5.02	5.63	4.63	5.09	Corn bran	30.15 B	29.72 B	27.16 AB	29.05 B
Wheat bran	18.48 Aa	15.68 Ba	10.09 b	14.86 B	Wheat bran	10.76	8.38 B	9.01 B	9.38 C	Wheat bran	5.43	6.08	5.96	5.83	Wheat bran	34.41 ABa	28.68 Bb	24.28 Bb	29.12 B
Corn bran + wheat bran	23.33 Aa	22.38 Aa	11.53 b	18.81 A	Corn bran + wheat bran	9.44 b	9.55 Bb	14.47 Aa	11.27 B	Corn bran + wheat bran	5.27	5.08	5.12	5.16	Corn bran + wheat bran	38.20 Aa	38.02 Aa	31.97 Ab	35.92 A
Mean	17.94 a	15.68 a	10.09 b		Mean	11.51	12.03	11.71		Mean	5.04	4.77	4.51		Mean	34.12 a	32.02 a	27.83 b	
C.V.	15.34				C.V.	26.02				C.V.	21.20				C.V.	16.94			

* The percentages of annatto residue incorporation (32.5%, 42.5%, and 52.5%) are expressed as *w*/*w* on a dry weight basis. Means followed by different uppercase letters within columns, and different lowercase letters within rows, differ significantly according to Tukey’s test (*p* ≤ 0.05).

**Table 3:** Biological efficiency (%) of *P. ostreatus* var. Florida cultivated on annatto residues under different supplementation strategies and residue proportions, for both field and industrial origins.

**Annatto field residues**				
**Supplementation**	% Annatto residue *			**Mean**
	**32.5**	**42.5**	**52.5**	
Corn bran	129.06	127.90 B	125.13 A	127.36 B
Wheat bran	135.41	244.91 Aa	113.19 ABb	165.51 A
Corn bran + wheat bran	144.16 a	125.92 Ba	88.48 Bb	119.52 B
Mean	136.24 b	166.24 a	108.93 c	
C.V.	18.48			
**Annatto industrial residues**				
**Supplementation**	**% Annatto residue**			**Mean**
	**32.5**	**42.5**	**52.5**	
Corn bran	111.66 Ba	78.21 Bb	71.49 Bb	88.00 B
Wheat bran	111.00 Ba	86.93 Bb	94.04 Ab	95.96 B
Corn bran + wheat bran	141.51 Aa	146.26 Aa	89.95 ABb	126.08 A
Mean	120.70 a	103.20 b	85.63 c	
C.V.	17.04			

* The percentages of annatto residue incorporation (32.5%, 42.5%, and 52.5%) are expressed as *w*/*w* on a dry weight basis. Means followed by different uppercase letters within columns, and different lowercase letters within rows, differ significantly according to Tukey’s test (*p* ≤ 0.05).

**Table 4:** Mean mushroom mass (g per bag) of *P. ostreatus* var. Florida cultivated on annatto residues under different supplementation strategies and residue proportions, for both field and industrial origins.

**Annatto field residues**				
**Supplementation**	% Annatto residue *			**Mean**
	**32.5**	**42.5**	**52.5**	
Corn bran	1.51 A	1.52 A	1.37 A	1.46 A
Wheat bran	1.24 B	1.21 B	1.22 AB	1.22 B
Corn bran + wheat bran	1.30 AB	1.28 AB	1.07 B	1.23 B
Mean	1.36	1.32	1.23	
C.V.	15.65			
**Annatto industrial residues**				
**Supplementation**	**% Annatto residue**			**Mean**
	**32.5**	**42.5**	**52.5**	
Corn bran	1.21	1.13	1.26	1.20
Wheat bran	1.21 ab	1.11 b	1.42 a	1.25
Corn bran + wheat bran	1.27	1.25	1.31	1.28
Mean	1.23 ab	1.16 b	1.33 a	
C.V.	14.65			

* The percentages of annatto residue incorporation (32.5%, 42.5%, and 52.5%) are expressed as *w*/*w* on a dry weight basis. Means followed by different uppercase letters within columns, and different lowercase letters within rows, differ significantly according to Tukey’s test (*p* ≤ 0.05).

**Table 5:** Number of mushrooms (per bag) of *P. ostreatus* var. Florida cultivated on annatto residues under different supplementation strategies and residue proportions, for both field and industrial origins.

**Annatto field residues**				
**Supplementation**	**% Annatto residue** *			**Mean**
	**32.5**	**42.5**	**52.5**	
Corn bran	169.11 Bb	173.85 Bab	213.00 Ba	186.24 B
Wheat bran	221.25 A	254.37 A	239.50 AB	238.35 A
Corn bran + wheat bran	253.12 Aab	215.40 Ab	261.75 Aa	241.26 A
Mean	212.68 b	216.24 ab	237.08 a	
C.V.	15.74			
**Annatto industrial residues**				
**Supplementation**	**% Annatto residue**			**Mean**
	**32.5**	**42.5**	**52.5**	
Corn bran	204.5 b	201.70 b	243.77 Aa	215.72 A
Wheat bran	211.62	197.00	170.22 B	192.04 B
Corn bran + wheat bran	229.33 a	213.66 a	147.50 Bb	198.73 AB
Mean	214.88 a	204.88 ab	188.69 b	
C.V.	17.73			

* The percentages of annatto residue incorporation (32.5%, 42.5%, and 52.5%) are expressed as *w*/*w* on a dry weight basis. Means followed by different uppercase letters within columns, and different lowercase letters within rows, differ significantly according to Tukey’s test (*p* ≤ 0.05).

**Table 6:** Mean bunch mass (g per bag) of *P. ostreatus* var. Florida cultivated on annatto residues under different supplementation strategies and residue proportions, for both field and industrial origins.

**Annatto field residues**				
**Supplementation**	**% Annatto residue** *			**Mean**
	**32.5**	**42.5**	**52.5**	
Corn bran	14.40	16.98 A	14.70 A	15.23 A
Wheat bran	10.76	10.71 B	9.02 B	10.17 B
Corn bran + wheat bran	11.20	13.51 AB	12.13 AB	12.23 B
Mean	12.21	13.59	12.06	
C.V.	26.67			
**Annatto industrial residues**				
**Supplementation**	**% Annatto residue**			**Mean**
	**32.5**	**42.5**	**52.5**	
Corn bran	9.33	9.87 A	10.29 A	9.81
Wheat bran	11.06 a	9.57 Aab	7.94 Bb	8.62
Corn bran + wheat bran	8.80 ab	6.55 Bb	10.30 Aa	9.58
Mean	9.75	8.78	9.57	
C.V.	21.49			

* The percentages of annatto residue incorporation (32.5%, 42.5%, and 52.5%) are expressed as *w*/*w* on a dry weight basis. Means followed by different uppercase letters within columns, and different lowercase letters within rows, differ significantly according to Tukey’s test (*p* ≤ 0.05).

**Table 7:** Number of bunches (per bag) of *P. ostreatus* var. Florida cultivated on annatto residues under different supplementation strategies and residue proportions, for both field and industrial origins.

**Annatto field residues**				
**Supplementation**	**% Annatto residue** *			**Mean**
	**32.5**	**42.5**	**52.5**	
Corn bran	19.77 B	15.57 B	20.55 B	18.88 C
Wheat bran	25.87 ABb	29.50 Aab	33.12 Aa	29.50 A
Corn bran + wheat bran	29.12 Aa	21.00 Bb	24.87 Bab	24.69 B
Mean	24.72	22.20	25.96	
C.V.	23.08			
**Annatto industrial residues**				
**Supplementation**	**% Annatto residue**			**Mean**
	**32.5**	**42.5**	**52.5**	
Corn bran	26.80 ab	23.30 b	30.34 a	26.68
Wheat bran	30.00 ab	33.37 a	24.00 b	28.92
Corn bran + wheat bran	27.22	27.88	25.12	26.80
Mean	27.88	27.81	26.53	
C.V.	18.99			

* The percentages of annatto residue incorporation (32.5%, 42.5%, and 52.5%) are expressed as *w*/*w* on a dry weight basis. Means followed by different uppercase letters within columns, and different lowercase letters within rows, differ significantly according to Tukey’s test (*p* ≤ 0.05).

## Data Availability

The data supporting the reported results are available in the Supplementary Material of this article. All experimental datasets and statistical analyses performed in this study are provided as Supplementary Files.

## Supplementary Materials

The following supporting information can be downloaded at https://www.mdpi.com/article/10.3390/microorganisms14071405/s1; Table S1: Complete ANOVA summaries for all evaluated variables in field and industrial annatto residue experiments, including degrees of freedom (DF), F-values, and *p*-values; Table S2: Complete raw dataset for all evaluated variables in the annatto residue cultivation experiments.
