# Supplementary material for: Improving Annatto Residue Bioconversion for Pleurotus ostreatus var. Florida Cultivation via Supplementation Strategies
Source: Microorganisms. 2026 Jun 25;14(7):1405. doi: 10.3390/microorganisms14071405 (PMC13413766; doi:10.3390/microorganisms14071405)
Supplement: Supplementary file 1 [file microorganisms-14-01405-s001.zip › microorganisms-4322211-supplementary.pdf]

Improving Annatto Residue Bioconversion for *Pleurotus ostreatus* var. Florida Cultivation via Supplementation Strategies

Milton Mineo Hirai, Lucas da Silva Alves, Wagner Gonçalves Vieira Junior,
Marcos Antônio Da Silva Freitas, Pedro Afonso Gomes Teixeira,
Adriano Taffarel Camargo De Paula, Isabela Vitória De Paula Moretti and Diego Cunha Zied

Supplementary Material

Table S1. Complete ANOVA summaries for all evaluated variables in field and industrial annatto residue experiments, including degrees of freedom (DF), F-values, and p-values.

Variable	Field residues				Industrial residues		
	Source of variation	*df*	*F-value*	*p-value*	*df*	*F-value*	*p-value*
1st flush yield	Annatto Residue (%)	2	0.574	0.565	2	20.29	**0.000**
	Supplementation	2	1.603	0.207	2	23.50	**0.000**
	Annatto Residue × Supplementation	4	0.994	0.415	4	3.73	**0.007**
	Residual	79			78		
2nd flush yield	Annatto Residue (%)	2	0.084	0.919	2	2.57	0.083
	Supplementation	2	7.216	**0.001**	2	18.39	**0.000**
	Annatto Residue × Supplementation	4	0.678	0.609	4	5.54	**0.000**
	Residual	79			78		
3rd flush yield	Annatto Residue (%)	2	1.523	0.224	2	0.380	0.685
	Supplementation	2	3.593	**0.032**	2	1.658	0.197
	Annatto Residue × Supplementation	4	0.719	0.581	4	0.432	0.784

Table S1. Complete ANOVA summaries for all evaluated variables in field and industrial annatto residue experiments, including degrees of freedom (DF), F-values, and p-values.

Variable	Field residues				Industrial residues		
	Source of variation	*df*	*F-value*	*p-value*	*df*	*F-value*	*p-value*
	Residual	79			78		
Total yield	Annatto Residue (%)	2	0.538	0.586	2	10.557	**0.001**
	Supplementation	2	1.574	0.213	2	15.661	**0.000**
	Annatto Residue × Supplementation	4	1.365	0.253	4	1.885	0.121
	Residual	79			78		
Biological efficiency	Annatto Residue (%)	2	36.376	**0.000**	2	28.835	**0.000**
	Supplementation	2	26.205	**0.000**	2	36.714	**0.000**
	Annatto Residue × Supplementation	4	24.154	**0.000**	4	8.569	**0.000**
	Residual	79			78		
Mean mushroom mass	Annatto Residue (%)	2	2.760	**0.070**	2	5.616	**0.005**
	Supplementation	2	11.439	**0.000**	2	1.429	0.246
	Annatto Residue × Supplementation	4	0.973	0.428	4	1.048	0.388

Table S1. Complete ANOVA summaries for all evaluated variables in field and industrial annatto residue experiments, including degrees of freedom (DF), F-values, and p-values.

Variable	Field residues				Industrial residues		
	Source of variation	*df*	*F-value*	*p-value*	*df*	*F-value*	*p-value*
	Residual	79			78		
Number of mushrooms	Annatto Residue (%)	2	3.558	**0.034**	2	3.551	**0.033**
	Supplementation	2	19.687	**0.000**	2	3.160	**0.048**
	Annatto Residue × Supplementation	4	3.492	**0.012**	4	7.758	**0.000**
	Residual	79			78		
Mean bunches mass	Annatto Residue (%)	2	1.625	0.204	2	1.744	0.182
	Supplementation	2	14.559	**0.000**	2	2.597	**0.081**
	Annatto Residue × Supplementation	4	0.805	0.524	4	5.625	**0.001**
	Residual	79			78		
Number of bunches	Annatto Residue (%)	2	2.919	**0.061**	2	0.560	0.573
	Supplementation	2	22.064	**0.000**	2	1.502	0.229
	Annatto Residue × Supplementation	4	3.416	**0.013**	4	5.762	**0.001**

Table S1. Complete ANOVA summaries for all evaluated variables in field and industrial annatto residue experiments, including degrees of freedom (DF), F-values, and p-values.

Variable	Field residues				Industrial residues		
	Source of variation	*df*	*F-value*	*p-value*	*df*	*F-value*	*p-value*
	Residual	79			78		

Table S2. Complete raw dataset for all evaluated variables in the annatto residue cultivation experiments.

Origin	Annato residue (%)	Supplementation	Number of mushrooms	Number of bunches	1st flush (kg)	1st flush (%)	2nd flush (kg)	2nd flush (%)	3rd flush (kg)	3rd flush (%)	Final Yield%	Substrate Moisture (%)	Weight of substrate (d.m)	Biological efficiency (BE) %
Field	35.5	WB	253	25	0,178	22,25	0,083	10,375	0,043	5,375	38	74	208	146,15
	35.5	WB	219	24	0,151	18,875	0,036	4,5	0,043	5,375	28,75	74	208	110,58
	35.5	WB	256	27	0,166	20,75	0,108	13,5	0,029	3,625	37,875	74	208	145,67
	35.5	WB	231	19	0,168	21	0,041	5,125	0,039	4,875	31	74	208	119,23
	35.5	WB	241	31	0,189	23,625	0,067	8,375	0,043	5,375	37,375	74	208	143,75
	35.5	WB	192	31	0,193	24,125	0,114	14,25	0,041	5,125	43,5	74	208	167,31
	35.5	WB	215	24	0,093	11,625	0,09	11,25	0,043	5,375	28,25	74	208	108,65
	35.5	WB	163	26	0,159	19,875	0,098	12,25	0,059	7,375	39,5	74	208	151,92
	35.5	WB	221	18	0,112	14	0,08	10	0,069	8,625	32,625	74	208	125,48
	42.5	WB	294	33	0,162	20,25	0,059	7,375	0,029	3,625	31,25	85	120	208,33
	42.5	WB	258	25	0,14	17,5	0,146	18,25	0,046	5,75	41,5	85	120	276,67
	42.5	WB	260	27	0,15	18,75	0,07	8,75	0,041	5,125	33,375	85	120	222,50
	42.5	WB	297	30	0,116	14,5	0,077	9,625	0,048	6	30,125	85	120	200,83
	42.5	WB	251	42	0,178	22,25	0,096	12	0,05	6,25	40,5	85	120	270,00
	42.5	WB	275	30	0,185	23,125	0,091	11,375	0,06	7,5	42	85	120	280,00
	42.5	WB	179	31	0,184	23	0,097	12,125	0,038	4,75	39,875	85	120	265,83
	42.5	WB	257	29	0,179	22,375	0,078	9,75	0,051	6,375	38,5	85	120	256,67
	42.5	WB	254	31	0,173	21,625	0,057	7,125	0,055	6,875	35,625	85	120	237,50
	42.5	WB	221	35	0,182	22,75	0,055	6,875	0,04	5	34,625	85	120	230,83
	52.5	WB	209	25	0,01	1,25	0,008	1	0,038	4,75	7	71	232	24,14
	52.5	WB	225	30	0,173	21,625	0,066	8,25	0,035	4,375	34,25	71	232	118,10
	52.5	WB	258	34	0,148	18,5	0,087	10,875	0,038	4,75	34,125	71	232	117,67
	52.5	WB	226	43	0,229	28,625	0,076	9,5	0,054	6,75	44,875	71	232	154,74
	52.5	WB	266	38	0,115	14,375	0,147	18,375	0,041	5,125	37,875	71	232	130,60
	52.5	WB	190	19	0,173	21,625	0,063	7,875	0,04	5	34,5	71	232	118,97
	52.5	WB	206	33	0,1335	16,6875	0,067	8,375	0,039	4,875	29,75	71	232	102,59

	52.5	WB	195	32	0,163	20,375	0,063	7,875	0,056	7	35,25	71	232	121,55
	52.5	WB	163	12	0,162	20,25	0,053	6,625	0,052	6,5	33,375	71	232	115,09
	52.5	WB	146	13	0,156	19,5	0,104	13	0,038	4,75	37,25	71	232	128,45
	35.5	CB	154	18	0,16	20	0,041	5,125	0,04	5	30,125	76	192	125,52
	35.5	CB	124	16	0,114	14,25	0,07	8,75	0,064	8	31	76	192	129,17
	35.5	CB	182	16	0,131	16,375	0,054	6,75	0,033	4,125	27,25	76	192	113,54
	35.5	CB	162	19	0,199	24,875	0,05	6,25	0,043	5,375	36,5	76	192	152,08
	35.5	CB	196	18	0,199	24,875	0,065	8,125	0,029	3,625	36,625	76	192	152,60
	35.5	CB	188	15	0,146	18,25	0,046	5,75	0,033	4,125	28,125	76	192	117,19
	35.5	CB	209	18	0,166	20,75	0,053	6,625	0,033	4,125	31,5	76	192	131,25
	35.5	CB	140	14	0,116	14,5	0,083	10,375	0,023	2,875	27,75	76	192	115,63
	35.5	CB	153	15	0,162	20,25	0,025	3,125	0,04	5	28,375	76	192	118,23
	35.5	CB	153	13	0,216	27	0,019	2,375	0,025	3,125	32,5	76	192	135,42
	42.5	CB	178	16	0,206	25,75	0,02	2,5	0,047	5,875	34,125	75	200	136,50
	42.5	CB	207	19	0,207	25,875	0,04	5	0,029	3,625	34,5	75	200	138,00
	42.5	CB	236	20	0,193	24,125	0,056	7	0,053	6,625	37,75	75	200	151,00
	42.5	CB	229	22	0,111	13,875	0,062	7,75	0,034	4,25	25,875	75	200	103,50
	42.5	CB	210	17	0,181	22,625	0,03	3,75	0,043	5,375	31,75	75	200	127,00
	42.5	CB	238	26	0,131	16,375	0,084	10,5	0,017	2,125	29	75	200	116,00
	42.5	CB	231	15	0,13	16,25	0,068	8,5	0,042	5,25	30	75	200	120,00
	42.5	CB	189	19	0,13	16,25	0,04	5	0,031	3,875	31,25	75	200	125,00
	42.5	CB	178	29	0,15	18,75	0,065	8,125	0,049	6,125	33	75	200	132,00
	42.5	CB	199	18	0,14	17,5	0,042	5,25	0,031	3,875	32,5	75	200	130,00
	52.5	CB	305	33	0,226	28,25	0,04	5	0,023	2,875	36,125	73	216	133,80
	52.5	CB	295	29	0,027	3,375	0,014	1,75	0,044	5,5	10,625	73	216	39,35
	52.5	CB	212	25	0,183	22,875	0,072	9	0,037	4,625	36,5	73	216	135,19
	52.5	CB	210	23	0,252	31,5	0,075	9,375	0,037	4,625	45,5	73	216	168,52
	52.5	CB	313	33	0,183	22,875	0,073	9,125	0,034	4,25	36,25	73	216	134,26
	52.5	CB	218	33	0,179	22,375	0,074	9,25	0,025	3,125	34,75	73	216	128,70
	52.5	CB	275	31	0,178	22,25	0,077	9,625	0,02	2,5	34,375	73	216	127,31
	52.5	CB	197	26	0,187	23,375	0,074	9,25	0,033	4,125	36,75	73	216	136,11
	52.5	CB	259	19	0,157	19,625	0,043	5,375	0,054	6,75	31,75	73	216	117,59
	52.5	CB	254	22	0,148	18,5	0,079	9,875	0,055	6,875	35,25	73	216	130,56
	35.5	CB+WB	277	17	0,2	25	0,057	7,125	0,038	4,75	36,875	73	216	136,57

	35.5	CB+WB	160	23	0,258	32,25	0,065	8,125	0,045	5,625	46	73	216	170,37
	35.5	CB+WB	253	30	0,209	26,125	0,055	6,875	0,05	6,25	39,25	73	216	145,37
	35.5	CB+WB	155	21	0,232	29	0,092	11,5	0,035	4,375	44,875	73	216	166,20
	35.5	CB+WB	176	19	0,127	15,875	0,081	10,125	0,046	5,75	31,75	73	216	117,59
	35.5	CB+WB	241	25	0,205	25,625	0,058	7,25	0,041	5,125	38	73	216	140,74
	35.5	CB+WB	148	19	0,283	35,375	0,048	6	0,075	9,375	50,75	73	216	187,96
	35.5	CB+WB	231	15	0,222	27,75	0,087	10,875	0,031	3,875	42,5	73	216	157,41
	35.5	CB+WB	251	21	0,074	9,25	0,047	5,875	0,041	5,125	20,25	73	216	75,00
	35.5	CB+WB	232	19	0,215	26,875	0,065	8,125	0,032	4	39	73	216	144,44
	42.5	CB+WB	241	17	0,157	19,625	0,098	12,25	0,041	5,125	37	73	216	137,04
	42.5	CB+WB	213	17	0,197	24,625	0,031	3,875	0,04	5	33,5	73	216	124,07
	42.5	CB+WB	267	22	0,176	22	0,099	12,375	0,054	6,75	41,125	73	216	152,31
	42.5	CB+WB	286	39	0,079	9,875	0,088	11	0,029	3,625	24,5	73	216	90,74
	42.5	CB+WB	301	34	0,187	23,375	0,078	9,75	0,04	5	38,125	73	216	141,20
	42.5	CB+WB	303	30	0,109	13,625	0,059	7,375	0,054	6,75	27,75	73	216	102,78
	42.5	CB+WB	253	25	0,133	16,625	0,074	9,25	0,027	3,375	29,25	73	216	108,33
	42.5	CB+WB	219	24	0,2	25	0,048	6	0,062	7,75	38,75	73	216	143,52
	42.5	CB+WB	256	27	0,148	18,5	0,046	5,75	0,042	5,25	29,5	73	216	109,26
	42.5	CB+WB	231	19	0,23	28,75	0,058	7,25	0,036	4,5	40,5	73	216	150,00
	52.5	CB+WB	241	31	0	0	0,043	5,375	0	0	5,375	63	296	14,53
	52.5	CB+WB	192	31	0,221	27,625	0,025	3,125	0,025	3,125	33,875	63	296	91,55
	52.5	CB+WB	215	24	0,178	22,25	0,078	9,75	0,021	2,625	34,625	63	296	93,58
	52.5	CB+WB	163	26	0,149	18,625	0,107	13,375	0,035	4,375	36,375	63	296	98,31
	52.5	CB+WB	221	18	0,148	18,5	0,049	6,125	0,043	5,375	30	63	296	81,08
	52.5	CB+WB	294	33	0,201	25,125	0,041	5,125	0,032	4	34,25	63	296	92,57
	52.5	CB+WB	258	25	0,179	22,375	0,067	8,375	0,065	8,125	38,875	63	296	105,07
	52.5	CB+WB	260	27	0,184	23	0,079	9,875	0,033	4,125	37	63	296	100,00
	52.5	CB+WB	297	30	0,198	24,75	0,043	5,375	0,036	4,5	34,625	63	296	93,58
	52.5	CB+WB	251	42	0,246	30,75	0,06	7,5	0,033	4,125	42,375	63	296	114,53
Industrial	35.5	WB	199	27	0,165	20,625	0,107	13,375	0,024	3	37	69	248	119,35
	35.5	WB	246	35	0,123	15,375	0,071	8,875	0,035	4,375	28,75	69	248	92,74
	35.5	WB	261	26	0,099	12,375	0,068	8,5	0,052	6,5	27,375	69	248	88,31
	35.5	WB	174	28	0,167	20,875	0,097	12,125	0,04	5	38	69	248	122,58
	35.5	WB	202	29	0,123	15,375	0,072	9	0,035	4,375	27,5	69	248	88,71

	35.5	WB	211	41	0,253	31,625	0,117	14,625	0,041	5,125	51,375	69	248	165,73
	35.5	WB	191	29	0,107	13,375	0,093	11,625	0,028	3,5	28,5	69	248	91,94
	35.5	WB	209	25	0,148	18,5	0,083	10,375	0,054	6,75	35,625	69	248	114,92
	35.5	WB	180	39	0,146	18,25	0,036	4,5	0,06	7,5	30,25	69	248	97,58
	35.5	WB	174	32	0,148	18,5	0,117	14,625	0,053	6,625	39,75	69	248	128,23
	42.5	WB	171	30	0,123	15,375	0,071	8,875	0,032	4	28,25	67	264	85,61
	42.5	WB	230	37	0,108	13,5	0,055	6,875	0,013	1,625	16,25	67	264	49,24
	42.5	WB	218	27	0,106	13,25	0,058	7,25	0,037	4,625	23,75	67	264	71,97
	42.5	WB	246	35	0,131	16,375	0,09	11,25	0,042	5,25	32,875	67	264	99,62
	42.5	WB	231	42	0,075	9,375	0,057	7,125	0,042	5,25	21,75	67	264	65,91
	42.5	WB	126	25	0,141	17,625	0,073	9,125	0,074	9,25	36	67	264	109,09
	42.5	WB	163	28	0,102	12,75	0,071	8,875	0,035	4,375	26	67	264	78,79
	42.5	WB	172	22	0,171	21,375	0,058	7,25	0,045	5,625	34,25	67	264	103,79
	42.5	WB	183	20	0,179	22,375	0,061	7,625	0,045	5,625	35,625	67	264	107,95
	42.5	WB	178	19	0,138	17,25	0,077	9,625	0,042	5,25	32,125	67	264	97,35
	52.5	WB	187	28	0,061	7,625	0,087	10,875	0,053	6,625	25,125	73	216	93,06
	52.5	WB	166	28	0,056	7	0,079	9,875	0,039	4,875	21,75	73	216	80,56
	52.5	WB	147	30	0,127	15,875	0,097	12,125	0,035	4,375	32,375	73	216	119,91
	52.5	WB	195	23	0,125	15,625	0,054	6,75	0,032	4	26,375	73	216	97,69
	52.5	WB	141	18	0,163	20,375	0,049	6,125	0,035	4,375	30,875	73	216	114,35
	52.5	WB	188	30	0	0	0,084	10,5	0,048	6	16,5	73	216	61,11
	52.5	WB	250	35	0,024	3	0,062	7,75	0,062	7,75	18,5	73	216	68,52
	52.5	WB	250	30	0,142	17,75	0,072	9	0,031	3,875	30,625	73	216	113,43
	52.5	WB	289	31	0,041	5,125	0,072	9	0,038	4,75	18,875	73	216	69,91
	52.5	WB	137	25	0,075	9,375	0,065	8,125	0,037	4,625	21,875	73	216	81,02
	35.5	CB	213	23	0,124	15,5	0,049	6,125	0,038	4,75	26,375	73	216	97,69
	35.5	CB	130	13	0,054	6,75	0,151	18,875	0,054	6,75	32,375	73	216	119,91
	35.5	CB	259	30	0,102	12,75	0,108	13,5	0,034	4,25	30,5	73	216	112,96
	35.5	CB	152	23	0,075	9,375	0,114	14,25	0,031	3,875	27,5	73	216	101,85
	35.5	CB	177	28	0,109	13,625	0,112	14	0,037	4,625	32,25	73	216	119,44
	35.5	CB	211	20	0,088	11	0,108	13,5	0,053	6,625	31,125	73	216	115,28
	35.5	CB	162	26	0,117	14,625	0,093	11,625	0,035	4,375	30,625	73	216	113,43
	35.5	CB	184	27	0,13	16,25	0,085	10,625	0,028	3,5	30,375	73	216	112,50
	35.5	CB	179	17	0,099	12,375	0,071	8,875	0,06	7,5	28,75	73	216	106,48

	35.5	CB	246	29	0,107	13,375	0,114	14,25	0,032	4	31,625	73	216	117,13
	42.5	CB	174	21	0,089	11,125	0,123	15,375	0,026	3,25	29,75	62	304	78,29
	42.5	CB	231	24	0,063	7,875	0,146	18,25	0,037	4,625	30,75	62	304	80,92
	42.5	CB	197	24	0,104	13	0,142	17,75	0,022	2,75	33,5	62	304	88,16
	42.5	CB	213	19	0,031	3,875	0,091	11,375	0,049	6,125	21,375	62	304	56,25
	42.5	CB	220	26	0,052	6,5	0,155	19,375	0,097	12,125	38	62	304	100,00
	42.5	CB	280	35	0,073	9,125	0,076	9,5	0,034	4,25	22,875	62	304	60,20
	42.5	CB	276	30	0,062	7,75	0,146	18,25	0,052	6,5	32,5	62	304	85,53
	42.5	CB	305	38	0,059	7,375	0,11	13,75	0,044	5,5	26,625	62	304	70,07
	42.5	CB	216	23	0,094	11,75	0,118	14,75	0,045	5,625	32,125	62	304	84,54
	52.5	CB	241	23	0,078	9,75	0,115	14,375	0,01	1,25	25,375	62	304	66,78
	52.5	CB	258	37	0,081	10,125	0,064	8	0,04	5	23,125	62	304	60,86
	52.5	CB	225	31	0,062	7,75	0,106	13,25	0,048	6	27	62	304	71,05
	52.5	CB	196	24	0,091	11,375	0,097	12,125	0,023	2,875	26,375	62	304	69,41
	52.5	CB	197	32	0,059	7,375	0,129	16,125	0,036	4,5	28	62	304	73,68
	52.5	CB	237	27	0,061	7,625	0,142	17,75	0,048	6	31,375	62	304	82,57
	52.5	CB	221	35	0,07	8,75	0,105	13,125	0,037	4,625	26,5	62	304	69,74
	52.5	CB	260	31	0,066	8,25	0,123	15,375	0,034	4,25	27,875	62	304	73,36
	52.5	CB	220	22	0,037	4,625	0,136	17	0,058	7,25	28,875	62	304	75,99
	35.5	CB+WB	283	25	0,193	24,125	0,051	6,375	0,044	5,5	36	73	216	133,33
	35.5	CB+WB	181	28	0,248	31	0,059	7,375	0,03	3,75	42,125	73	216	156,02
	35.5	CB+WB	240	29	0,211	26,375	0,054	6,75	0,028	3,5	36,625	73	216	135,65
	35.5	CB+WB	251	31	0,196	24,5	0,105	13,125	0,057	7,125	44,75	73	216	165,74
	35.5	CB+WB	171	17	0,168	21	0,085	10,625	0,052	6,5	38,125	73	216	141,20
	35.5	CB+WB	285	26	0,162	20,25	0,074	9,25	0,039	4,875	34,375	73	216	127,31
	35.5	CB+WB	219	27	0,21	26,25	0,059	7,375	0,063	7,875	41,5	73	216	153,70
	35.5	CB+WB	186	27	0,194	24,25	0,068	8,5	0,023	2,875	35,625	73	216	131,94
	35.5	CB+WB	179	23	0,098	12,25	0,125	15,625	0,055	6,875	34,75	73	216	128,70
	42.5	CB+WB	172	25	0,18	22,5	0,049	6,125	0,055	6,875	35,5	74	208	136,54
	42.5	CB+WB	271	37	0,131	16,375	0,097	12,125	0,042	5,25	33,75	74	208	129,81
	42.5	CB+WB	209	28	0,176	22	0,065	8,125	0,06	7,5	37,625	74	208	144,71
	42.5	CB+WB	212	26	0,193	24,125	0,081	10,125	0,049	6,125	40,375	74	208	155,29
	42.5	CB+WB	190	32	0,104	13	0,121	15,125	0,059	7,375	35,5	74	208	136,54
	42.5	CB+WB	180	28	0,313	39,125	0,077	9,625	0,045	5,625	54,375	74	208	209,13

	42.5	CB+WB	141	31	0,15	18,75	0,065	8,125	0,032	4	30,875	74	208	118,75
	42.5	CB+WB	138	24	0,149	18,625	0,09	11,25	0,055	6,875	36,75	74	208	141,35
	42.5	CB+WB	135	20	0,216	27	0,043	5,375	0,041	5,125	37,5	74	208	144,23
	52.5	CB+WB	146	24	0,074	9,25	0,075	9,375	0,048	6	24,625	66	272	72,43
	52.5	CB+WB	163	33	0,095	11,875	0,135	16,875	0,041	5,125	33,875	66	272	99,63
	52.5	CB+WB	125	18	0,096	12	0,131	16,375	0,053	6,625	35	66	272	102,94
	52.5	CB+WB	152	23	0,135	16,875	0,09	11,25	0,035	4,375	32,5	66	272	95,59
	52.5	CB+WB	199	27	0,067	8,375	0,138	17,25	0,039	4,875	30,5	66	272	89,71
	52.5	CB+WB	246	35	0,089	11,125	0,09	11,25	0,049	6,125	28,5	66	272	83,82
	52.5	CB+WB	261	26	0,097	12,125	0,11	13,75	0,048	6	31,875	66	272	93,75
	52.5	CB+WB	174	28	0,056	7	0,12	15	0,049	6,125	28,125	66	272	82,72
	52.5	CB+WB	202	29	0,147	18,375	0,136	17	0,051	6,375	41,75	66	272	122,79
	52.5	CB+WB	211	41	0,067	8,375	0,133	16,625	0,064	8	33	66	272	97,06

Tabla S2.
